# Soil Microbiome Response to Contamination with Bisphenol A, Bisphenol F and Bisphenol S

**DOI:** 10.3390/ijms21103529

**Published:** 2020-05-16

**Authors:** Magdalena Zaborowska, Jadwiga Wyszkowska, Agata Borowik

**Affiliations:** Department of Microbiology, University of Warmia and Mazury in Olsztyn, 10-727 Olsztyn, Poland; m.zaborowska@uwm.edu.pl (M.Z.); agata.borowik@uwm.edu.pl (A.B.)

**Keywords:** BPA, BPF, BPS, soil enzymes, soil microorganisms, biodiversity

## Abstract

The choice of the study objective was affected by numerous controversies and concerns around bisphenol F (BPF) and bisphenol S (BPS)—analogues of bisphenol A (BPA). The study focused on the determination and comparison of the scale of the BPA, BPF, and BPS impact on the soil microbiome and its enzymatic activity. The following parameters were determined in soil uncontaminated and contaminated with BPA, BPF, and BPS: the count of eleven groups of microorganisms, colony development (CD) index, microorganism ecophysiological diversity (EP) index, genetic diversity of bacteria and activity of dehydrogenases (Deh), urease (Ure), catalase (Cat), acid phosphatase (Pac), alkaline phosphatase (Pal), arylsulphatase (Aryl) and *β*-glucosidase (Glu). Bisphenols A, S and F significantly disrupted the soil homeostasis. BPF is regarded as the most toxic, followed by BPS and BPA. BPF and BPS reduced the abundance of *Proteobacteria* and *Acidobacteria* and increased that of *Actinobacteria*. Unique types of bacteria were identified as well as the characteristics of each bisphenol: *Lysobacter, Steroidobacter, Variovorax, Mycoplana,* for BPA, *Caldilinea, Arthrobacter, Cellulosimicrobium and Promicromonospora* for BPF and *Dactylosporangium Geodermatophilus, Sphingopyxis* for BPS. Considering the strength of a negative impact of bisphenols on the soil biochemical activity, they can be arranged as follows: BPS > BPF > BPA. Urease and arylsulphatase proved to be the most susceptible and dehydrogenases the least susceptible to bisphenols pressure, regardless of the study duration.

## 1. Introduction

Twentieth-century chemical synthesis created conditions for the production of plasticisers, and in consequence, of their components [1]. The most hazardous of these include dioxins [2] and acrylonitrile [3], as well as bisphenol A (BPA) [4]. Currently, alternative substances are being sought to alleviate concerns associated with bisphenol. Sixteen BPA analogues have been used in the chemical industry with bisphenol S (BPS) and bisphenol F (BPF) being its chief substitutes [1,5]. According to Molina-Molina et al. [6], the toxicity of the bisphenols decreases with the increasing polarity of their molecules. Bisphenol F (BPF) is obtained in the process of phenol hydroxyalkylation and formaldehyde catalysis. The catalysis runs with a Brönsted’s ionic liquid [7] or aluminosilicate catalysts MCM-41 [8]. Bisphenol F (BPF) differs from BPA by the absence of two methyl groups bound to the central carbon atom of its molecule that results in its lower polarity. On the other hand, because of the presence of two phenolic groups on each side of the sulphonic group, BPS has a similar chemical structure to BPA, but higher thermal stability [9,10]. Bisphenol A (BPA) is formed by condensation of an acetone molecule and two molecules of phenol, catalysed by hydrogen chloride or an ion-exchange resin [11].

Except for BPF, bisphenols are covered by the Community Rolling Action Plan (CoRAP) [12]. According to the data from the European Chemicals Agency (ECHA 2020), the annual production output or import of BPA and BPS now amount to 1–10 10^9^ kg. According to Cydzik-Kwiatkowska et al. [13], the constant level of BPA production output is probably associated with the widespread use of its analogues in resin and polycarbonate coatings. An increase in BPF production output may be caused by higher resistance of the BPF epoxide resin to solvents than of the BPA resin [14]. Secondary environment contamination by BPS is caused by bisphenol adsorption on PVC microplastics, which is affected by hydrogen and halogen bonds [15]. The bisphenols are widely used in the manufacturing of adhesives, varnishes, flooring, dentist sealing and thermal paper [5,16]. Interestingly, pursuant to Annex XVII of REACH (ECHA 2020), marketing thermal paper containing at least 0.2% w/w of BPA has not been allowed since 2 January 2020 [12]. Legal regulations prohibit the use of BPA in bottles for infants and in the inner lining of food containers, and their tolerable daily intake (TDI) caused by BPA migration from cans and containers to food is increasingly strictly restricted and is now 4 μg kg^−1^ body weight ( b.w.) d^−1^ [17,18].

New regulations have been passed due to the fact of reports on the toxic effect of bisphenols on the human body. Bisphenol A (BPA), bisphenol S (BPS) and bisphenol F (BPF) are regarded as an endocrine-disrupting chemical (EDC) with confirmed endocrine potential [19,20]. BPA is strongly bound with oestrogen receptors (ERα and ERβ) and γ (ERR-γ) and, to a lesser extent, with androgen receptors (AR) [21]. However, BPF shows five times higher oestrogenicity than BPA in the human MCF-7 and HepG2 cells proliferation test [22]. Exposure to BPF significantly decreases biosynthesis of GSH, glutathione synthetase (Gss), y-glutamyltransferase (Ggt) and glutaminase (Gls2) which characterise the metabolome and lipidome of the liver and kidneys [20]. Moreover, BPF and BPS have a negative impact on 5α-reductase, which is the key enzyme responsible for neurosteroidogenase [23]. Numerous studies have documented cytotoxicity, neurotoxicity and genotoxicity of the bisphenols under study [6,24,25]. A correlation was also observed between the concentration of BPA, BPF and BPS and the concentration of 8-hydroxydeoxyguanosine (8-OHdG) in urine which is regarded as the oxidative marker of DNA damage [26]. Ho et al. [27] report that both BPS and BPA demonstrate the broadest spectrum of cancer-promoting effects as a result of the centrosome function and microtubule organization disruption.

Bisphenols are referred to as “priority substances”. This term takes into account a combination of toxicity and occurrence in the environment in consequence of infiltration of environmental matrices, such as the air, water and soil and refers to the Agency for Toxic Substances and Disease Registry’s Substances Priority List (ATSDR), which contains phenolic compounds [28]. Wang et al. [29] report that mean BP concentration in dust samples was the lowest in Pakistan (0.15 µg g^−1^ of dust) and the highest in Greece (4.48 µg g^−1^ of dust). Surface waters in the south-east of Asia were contaminated mainly with BPF [30], whereas waters in the USA, Germany and Italy were contaminated with BPA [5]. Huang et al. [31] reported detectable levels of BPS (ranging from 0.02 to 65.60 μg dm^−3^) in surface waters and sludge in the south of China. Furthermore, Yamazaki et al. [30] reported up to 7.2 μg BPS dm^−3^ in surface waters in India, which probably generated the largest mean annual increase in BPA production output in this country [32]. According to Danzl et al. [14], the biodegradability of bisphenols in seawater can be put in the following order: BPF > BPA > BPS. Bisphenols are the most stable in sludge (t1/2 = 135-1621 days), with t1/2 for BPS being 135 days, and for BPA 337.5 [5]. The highest concentrations in the surface deposits in the industrial areas of Japan, Korea and the USA reached 23.3 μg g^−1^ in deposits [33].

The bisphenol degradation rate in the soil is affected by abiotic and biotic factors, molecular features and microorganism diversity, physicochemical properties of soil, its pH and redox potential [34,35]. BPA, BPF and BPS were found in soil samples collected in arable land and soils of the industrial areas in Spain. According to Pérez et al. [36], they originate in post-sewage waters used for field irrigation. Manganese oxides are regarded as the strongest, natural oxidisers of phenolic compounds in soil [37]. BPS shows higher reactivity towards MnO_2_ than BPA [38]. Im et al. [39] identified 4-hydroxycumyl alcohol (HCA), a product of oxidation of BPA electrons by MnO_2_ which undergoes microbiological degradation faster than bisphenol itself. Bisphenol transformation in soil is greatly affected by iodide ion (I^−^), which is oxidised to hypoiodic acid (HOI) or molecular iodine (I2) [40]. Vikesland et al. [41] demonstrated that I^−^ stimulates transformations of bisphenols, triclosan and phenol. A better understanding of these interactions enables optimisation of the effects of environmental contamination with bisphenols. It should be noted that the bisphenol stability in soil t1/2 = 30–360 days, BPS—30 days and BPA—75 days [5]. Bisphenol transformation is also affected by polyphenols, mainly flavonoids, which are among the most commonly occurring natural compounds in the plant world [42]. Bisphenol antagonists in plants include resveratrol (which inhibits the formation of the Erα-ligand complex and blocks the receptor interaction with the insulin-like growth factor-1 receptor (IGF-1R) induced by BPA [43]) and kaempferol, which promotes apoptosis of the Michigan Cancer Foundation-7 (MCF-7) treated with bisphenol [44].

According to the International Patent Classification (IPC), bacteria have the highest bioremediation potential towards bisphenols [45]. Moreover, enzymes are critical parameters, components of modelling and simulation methods that determine the soil fertility [46,47]. The most effective enzymes include oxidoreductases such as: lignin peroxidase (LiP) (EC 1.11.1.14), manganese-dependent peroxidase (MnP) (EC 1.11.1.13), laccase (EC 1.10.3.2) and tyrosinase (EC 1.14.18.1) [48,49]. According to Gassara et al. [50] LiP, MnP and laccases were able to degrade BPA by 90%. LiP was isolated from the fungus *Penicillium chrysosporium*, MnP from *Ganoderma lucidum* IBL-05, and laccases from *Trametes versicolor* and *Cerrena unicolor* [51,52]. Most recent studies indicate that *Pseudomonas putida* and *Pseudomonas stutzeri* exhibit significant biodegradation capacity towards phenols [53,54]. Particularly noteworthy is the group of genetically modified bacteria (GMMs), among others: *Comanonas testosteroni* E23, *Pseudomonas putida* F1-Te, *Pseudomonas putida* DOT-T1-Km, *Pseudomonas* sp. B13 [54,55]. An example of a microorganism having a set of genes encoding enzymes involved in the degradation of many phenol derivatives within one cell is *Cupriavidus necator* JMP134 [56].

Since for a full characterisation of microorganisms it is necessary to know the nucleotide sequence in their genomes, a metagenomic analysis is an alternative to environmental microbiology, which creates an opportunity for a holistic analysis of the soil microbiome structural diversity [57]. However, knowledge of the mechanisms of bisphenol toxicity to microorganisms is limited. Rasheed et al. [58] suggest that bisphenols bind to bacterial cell membranes by intercalation, disrupt their activity and block lipid synthesis. Biodegradation arising from bisphenol use as an energy source for microorganisms was identified mainly for BPA, with two mechanisms as its matrix [59]. One involves an oxidative transfer of a methyl group within the BPA molecule [60]. The other involves hydroxylation of one or two phenolic rings following the aromatic ring cleavage [61].

The lack of extensive data regarding the determination of the comparative scale of homeostasis disruption for soil exposed to BPA and its analogues—BPF and BPS—provided encouragement to analyse the soil microbiome response to soil contamination with bisphenols. This is a continuation of a research procedure and it is a necessary step in expanding and systematising the knowledge of differences in phenolic compound toxicity, not only with respect to its microbiological activity and biodiversity, but also soil enzyme sensitivity to different bisphenols, which, so far, has received limited attention on the global scale.

## 2. Results

Selected physico-chemical, chemical and biochemical properties of the soil was characterized in the experiment and presented in Table 1. 

### 2.1. Counts and Diversity of Microorganisms

**Figure 1:**
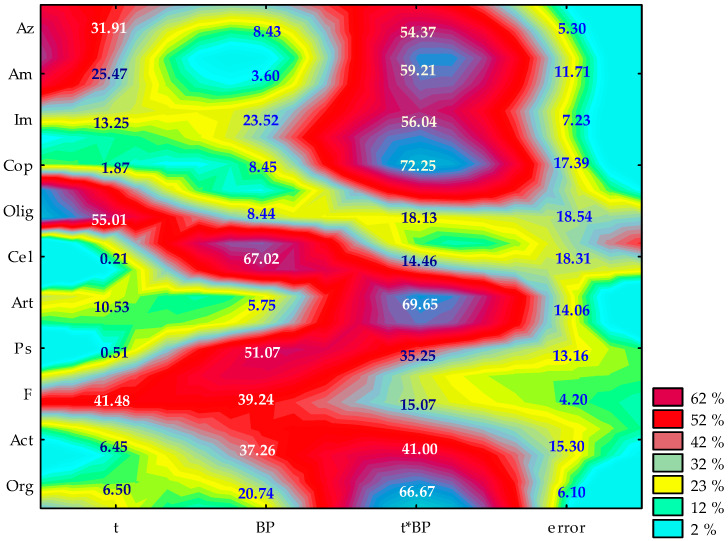
The share of independent variables in the evolution of the microorganisms activity (η^2^): t—time, BP—kind of bisphenol; time*kind of bisphenol; Org—organotrophic bacteria, Act—Actinomycetes, F—mould fungi, Ps—*Pseudomonas* sp., Art—*Arthrobacter* sp., Cel—cellulolytic bacteria, Olig—oligotrophic bacteria, Cop—copiotrophic bacteria, Im—nitrogen immobilizing bacteria, Am—ammonification bacteria, Az—*Azotobacter* sp. (two-way analysis of variance, ANOVA, at *p* < 0.05).

The microorganism count is significantly affected by the time of exposure to bisphenols and by the type of the substance used (Figure 1). The time had the greatest impact on the count of oligotrophic bacteria (51%) and fungi (41.48%), whereas the type of bisphenol had the greatest impact on the count of the cellulolytic bacteria (67.02%) and *Pseudomonas* sp. (51.07%). Bisphenol A (BPA) modified the soil microorganism growth. Bisphenol stimulated an increase in the count of all groups of microorganisms except for the cellulolytic bacteria and *Azotobacter* sp. on day 30 of the experiment (Table 2).

The opposite trends were observed on day 15. The microorganism response to soil contamination with BPF was much more varied. Regardless of the time of exposure to BPF, the organotrophic and cellulolytic bacteria count was decreased by this bisphenol. Moreover, the nitrogen immobilising bacteria and *Azotobacter* sp. count was found to decrease on day 15 of the experiment and that of copiotrophic, ammonification bacteria, and *Arthrobacter* sp. on day 30 of the experiment. The application of BPF to soil had the most beneficial effect on fungi, *Pseudomonas* sp. and oligotrophic bacteria. On the other hand, BPS had a stronger inhibitory impact on the soil microbiome on day 15 of the experiment. It reduced the count of *Azotobacter* sp., cellulolytic, nitrogen immobilising, organotrophic and oligotrophic bacteria by 77.18%; 64.77%; 62.34%; 47.16% and 8.34%, respectively. After 30 days of the soil incubation, stimulation of the count of microorganisms exposed to BPS was observed, considering an increasing count of all the microorganism groups under study, except *Arthrobacter* sp., cellulolytic and copiotrophic bacteria.

**Figure 2:**
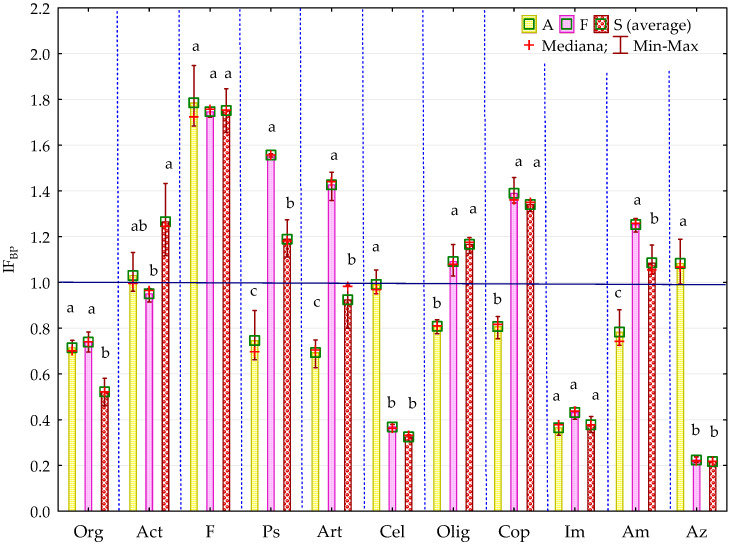
Coefficient of impact (IFBP) of bisphenols for group of microorganisms in soil contaminated with BPA, BPF and BPS on the 15th day of research, A—soil contaminated with BPA; F—soil contaminated with BPF; S—soil contaminated with BPS; Homogeneous groups denoted with letters (a–c) were calculated separately for each group of microorganisms (for abbreviations see Figure 1).

**Figure 3:**
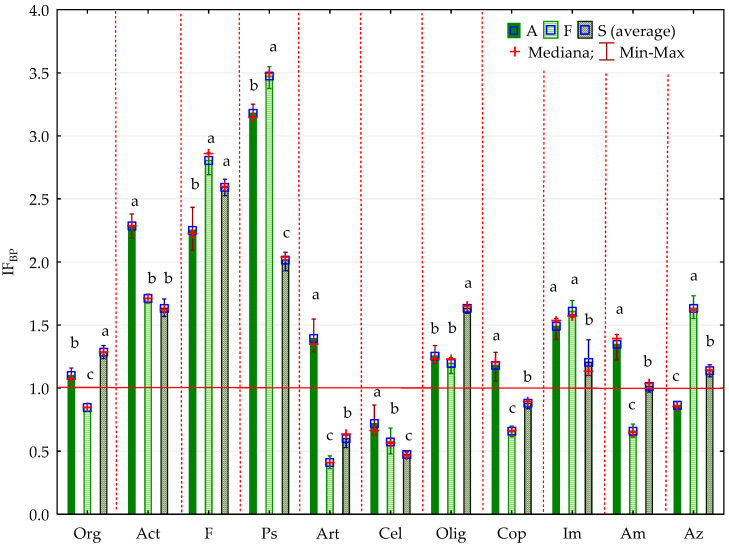
Coefficient of impact (IF_BP_) of bisphenols for group of microorganisms in soil contaminated with BPA, BPF and BPS on the 30th day of research; Homogeneous groups denoted with letters (a–c) were calculated separately for each group of microorganisms (for abbreviations see Figure 1).

The bisphenol impact factor (IF_BP_) confirmed the sensitivity of organotrophic and cellulolytic bacteria to pressure from BPA, BPF and BPS. It also provides grounds for the claim that BPF is the most toxic phenolic compound, followed by BPS, and the impact of BPA on the microorganism count is the weakest (Figure 2, Figure 3).

**Figure 4:**
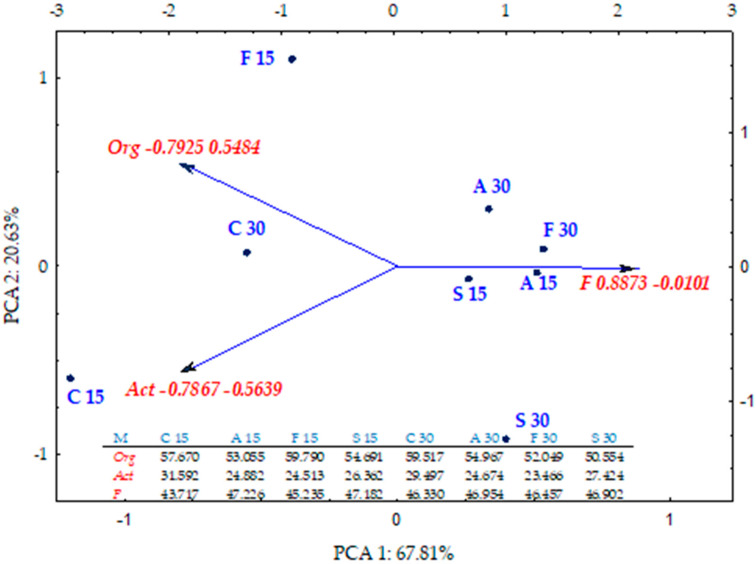
Colony development index (CD) for Org—organotrophic bacteria, Act—Actinomycetes and F—fungi in soil contaminated BPA, BPF and BPS on the 15th and 30th day of research—PCA method; M—groups of microorganisms; C—control; A—soil contaminated with BPA; F—soil contaminated with BPF; S—soil contaminated with BPS.

**Figure 5:**
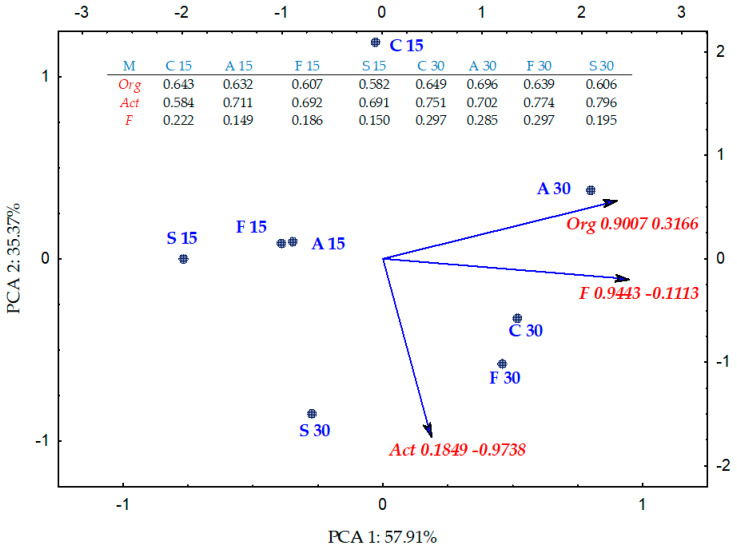
Ecophysiological diversity factor (EP) for Org—organotrophic bacteria, Act—Actinomycetes and F—fungi in soil contaminated BPA, BPF and BPS on the 15th and 30th day of research—PCA method; M—groups of microorganisms; C—control; A—soil contaminated with BPA; F—soil contaminated with BPF; S—soil contaminated with S.

Valuable information on the scale of disruption of the microbiological balance in soil exposed to bisphenol contamination and the complexity of the processes that initiate them is provided by the colony development index (CD) (Figure 4) and the ecophysiological diversity index (EP) (Figure 5). These indices were correlated with the phenolic compound type and the soil incubation time. The differences in the impact of individual bisphenols were demonstrated by multidimensional PCA analysis (Figure 4). The first principal component (PCA1) explaining 67.81% of the total data variance generated a positive value of the primary variable vector only for fungi (0.887). The case dislocation emphasised the fungi colony development stimulation by BPA, BPF and BPS. The tendency was further made more precise by the PCA analysis. The highest CD for organotrophic bacteria suggests that this group is dominated by fast replicating microorganisms. Nevertheless, the application of bisphenols to soil was not as effective in accelerating the development of organotrophic bacteria as it was for fungi, either on day 15 or day 30 of the experiment. The PCA also revealed that Actinobacteria are slowly growing organisms, regardless of whether or not they were exposed to bisphenols. However, unlike for fungi, the ecophysiological diversity index (EP) for this group of microorganisms reached the highest values (Figure 5).

The position of all cases on the PCA plot shows that all the bisphenols reduced the ecophysiological diversity of organotrophic bacteria and fungi on day 15 of the experiment and the impact of BPS was the greatest. The negative impact of this bisphenol persisted until day 30, while the inhibitory strength of the other bisphenols diminished. The relationships revealed made more credible the corresponding standardised vectors of primary variables with positive values relative to the first principal component (PCA1) for all the microorganism groups and negative for Act (−0.974) relative to the second principal component (PCA2) describing 33.37% of the variance of variables.

**Figure 6:**
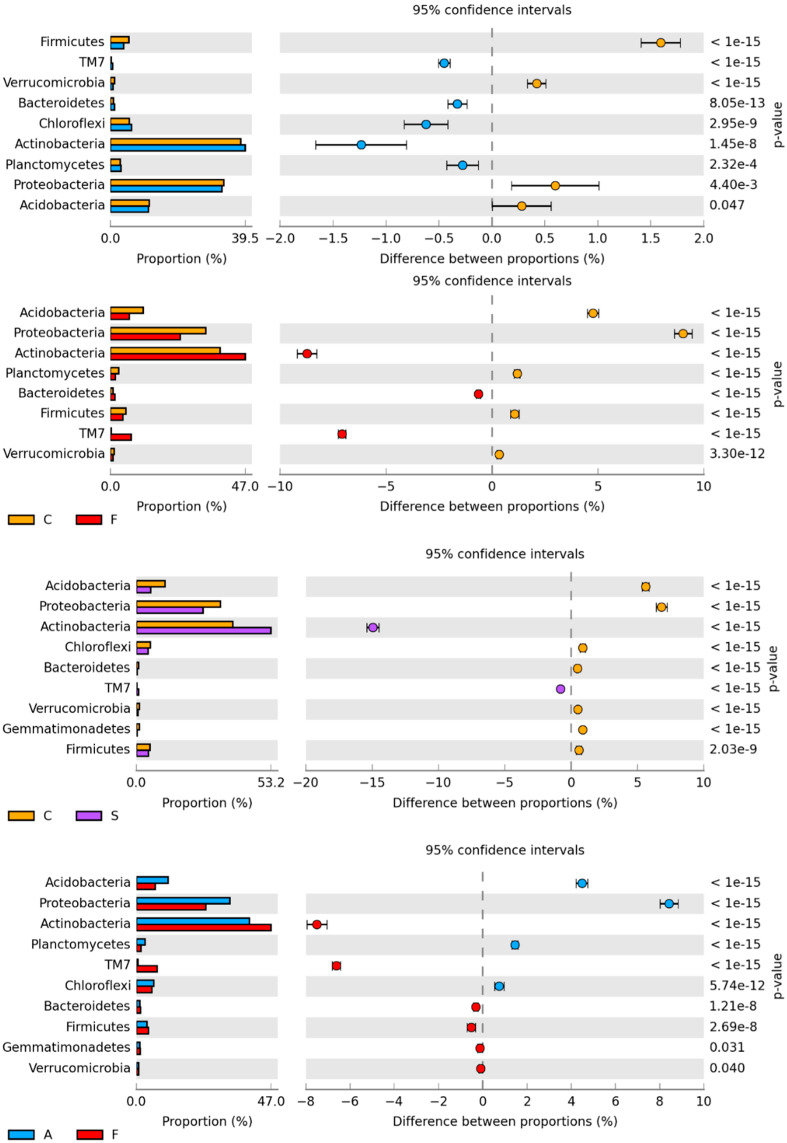

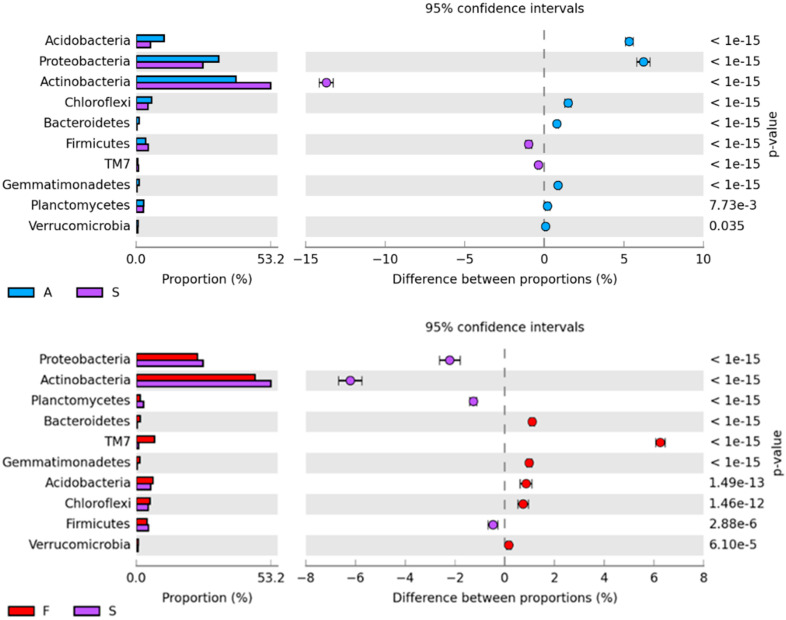
The relative abundance of the dominant bacterial type in soil with a difference between proportions ≥ 1%. C—uncontaminated soil; A—soil contaminated with BPA; F—soil contaminated with BPF; S—soil contaminated with BPS.

The greatest number of OTU among all the identified phyla was observed in *Actinobacteria* and Proteobacteria (Figure 6). Actinobacteria accounted for 37.5% of all bacteria in soil uncontaminated with bisphenols and 39.0% in soil contaminated with BPA, 46.4%—with BPF and 52.7%—with BPS. *Proteobacteria* accounted for 32.6% (control), 32.2% (BPA), 23.9% (BPF) and 26.2% (BPS). Bisphenol F (BPF) and bisphenol S (BPS) increased the abundance of *Actinobacteria* by 8.9% and 15.2%, respectively, compared to the control, whereas they reduced the abundance of *Proteobacteria* (BPS by 8.7% and BPF by 6.4%) and *Acidobacteria* (BPS by 4.6% and BPF by 5.5%). Conversely, BPA did not have a significant impact on these types. Only BPF had a positive impact on TM7.

**Figure 7:**
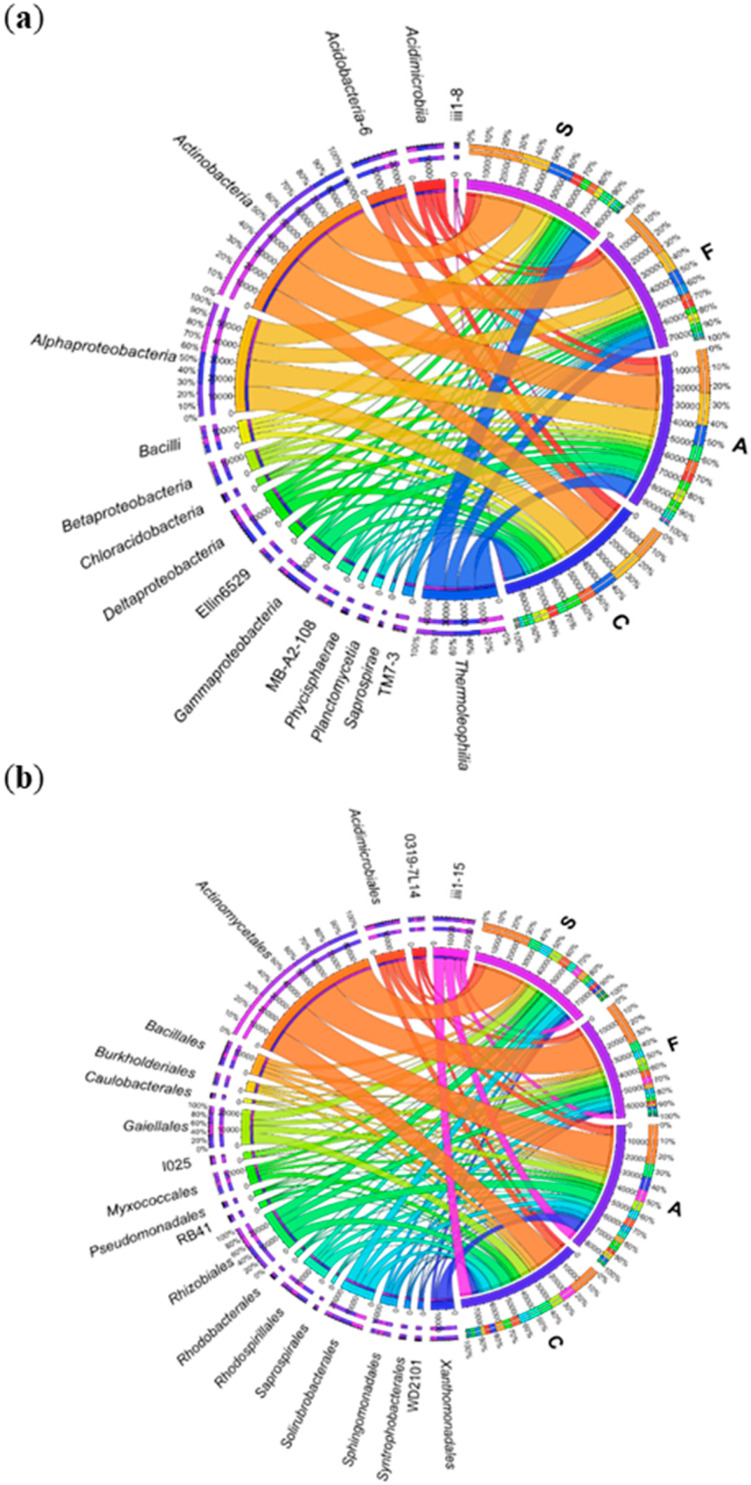
The relative abundance of dominant classes (**a**) and orders (**b**) of bacteria in soil with a difference between proportions ≥ 1%. C—uncontaminated soil; A—soil contaminated with BPA; F—soil contaminated with BPF; S—soil contaminated with BPS.

According to the number of OTU at the class level, the most representative were *Actinobacteria* and *Thermoleophilia* of phylum *Actinobacteria*, and *Alphaproteobacteria* of phylum *Proteobacteria* (Figure 7a). The highest (and the most diverse) number of OTUs for individual objects was identified within the *Actinobacteria* class: control—16,511 OTU, BPA—22,715 OTU, BPF—24,481 OTU, BPS—26,760 OTU. Interestingly, BPF generated a large number of OTU in classes of *Saprospirae* and TM7-3, and BPA: *Phycisphaerae*, iii1-8, *Chloracidobacteria*. It also induced an increase in this parameter for *Gammaproteobacteria*, whereas BPF and BPS reduced it. The impact of bisphenols on bacteria classes also affected the lower taxon, i.e., order (Figure 7b). *Actinomycetales* of Phylum *Acidobacteria* was the dominant order in this study. Its abundance was increased all the bisphenols. Subsequent orders in terms of their abundance in this Phylum: *Gaiellales*, iii1–15, *Solirubrobacterales* and *Acidimicrobiales*. Furthermore, Phylum *Proteobacteria* were dominated by orders *Rhizobiales* and *Rhodospirillales*. All the bisphenols reduced the OTU number for bacteria of the orders *Rhodospirillales*, and the OTU number in the order *Rhizobiales*—only BPF and BPS.

**Figure 8:**
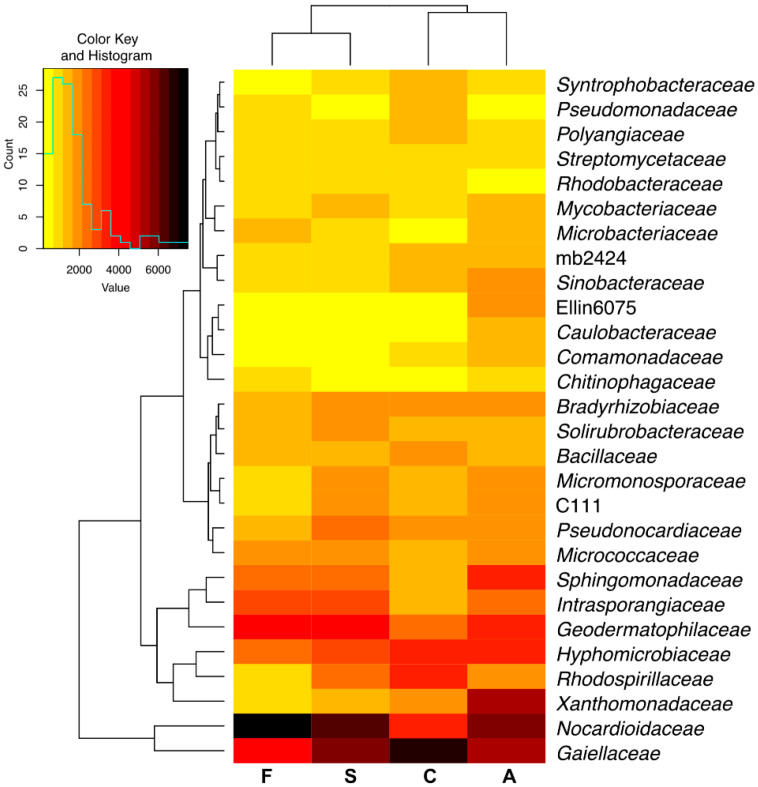
Heat map and associations of the number of bacterial families in soil with a difference between proportions ≥ 1%. C—uncontaminated soil; A—soil contaminated with BPA; F—soil contaminated with BPF; S—soil contaminated with BPS.

The lower taxon was dominated by *Gaiellaceae*, *Nocardioidaceae* and *Geodermatophilaceae* of phylum *Actinobacteria* and *Hyphomicrobiaceae* and *Xanthomonadaceae* of phylum *Proteobacteria* (Figure 8). All bisphenols reduced the abundance of OTUs of the following bacteria: *Gaiellaceae, Rhodospirillaceae, Bacillaceae, Syntrophobacteraceae, Pseudomonadaceae, Polyangiaceae*, *Rhodobacteraceae*, and increased that of *Nocardioidaceae, Geodermatophilaceae, Intrasporangiaceae, Sphingomonadaceae, Microbacteriaceae and Micrococcaceae*. The microbiological balance on the family level was caused to a lesser extent by BPA than by BPS and BPF.

**Figure 9:**
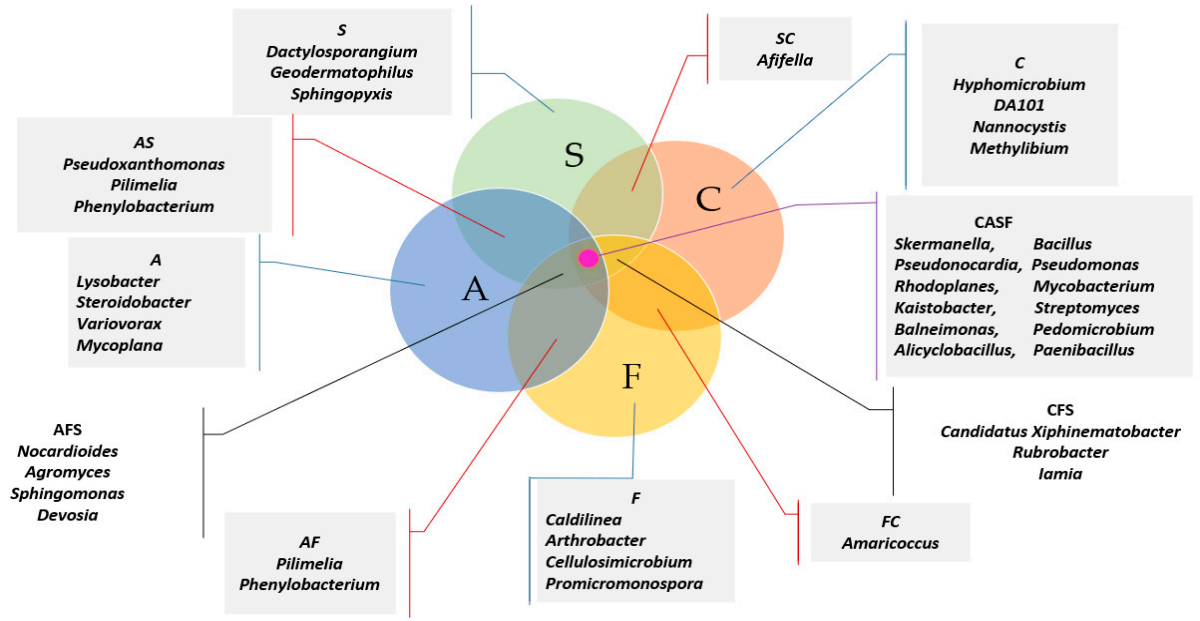
Venn diagram showing unique and common types of bacteria, based on all OTU data. C—uncontaminated soil; A—soil contaminated with BPA; F—soil contaminated with BPF; S—soil contaminated with BPS.

The Venn analysis helped to identify unique bacteria types characteristic of individual study objects (Figure 9). In the control soil, those included: *Hyphomicrobium*, DA101, *Nitrospira, Nannocystis*, *Methylibium*, in that contaminated with BPA: *Lysobacter, Steroidobacter, Variovorax, Mycoplana*, in that contaminated with BPF: *Caldilinea, Arthrobacter, Cellulosimicrobium* and *Promicromonospora* and in that contaminated with BPS: *Dactylosporangium, Geodermatophilus* and *Sphingopyxis*. Twelve genera common to all the objects were identified along with the native genera. It is notable that all the genera in the core microbiome in the soil contaminated with BPA were of Phylum *Proteobacteria*, whereas in the soil contaminated with BPF 3 the genera belong to *Actinobacteria* and 1 to *Chloroflexi*, and in soil contaminated with BPS—2 genera belong to Actinobacteria and 1—to *Proteobacteria*.

### 2.2. Enzyme Activity

**Figure 10:**
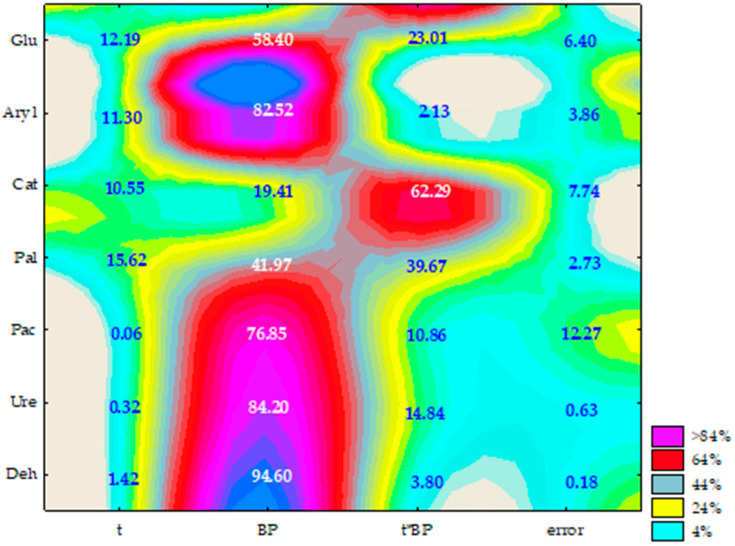
The share of independent variables in the evolution of the soil enzyme activity (η^2^): t—time, BP—kind of bisphenol; time*kind of bisphenol; Deh—dehydrogenases; Ure—urease, Pal—alkaline phosphatase, Pac—acid phosphatase, Aryl - arylsulphatase, Glu—*β*-glucosidase, (two-way analysis of variance, ANOVA, at *p* < 0.05).

The study results showed that all three bisphenols, bisphenol A, bisphenol F and bisphenol S, modified the soil biochemical activity significantly, although to a different extent. This is evidenced by the η^2^ values for each enzyme, which can be put in the following order: Deh > Ure > Aryl > Pac > Glu > Pal > Cat (Figure 10).

Time affected the activity of Pal (15.62%), Glu (12.19%) and Aryl (11.30%) to the greatest extent. Contrary to expectations, BPA was found to stimulate the activity of Deh, both on day 15 and on day 30 of the experiment (Table 3). The enzyme activity increased 2 and 3 fold compared to the control objects. Bisphenol A (BPA) also stimulated the activity of Pal and, to a lesser extent Pac, regardless of the experiment duration, which also corresponds to the homogeneous groups identified in the study. Bisphenol A (BPA) had an inhibitory effect on the activity of Cat, Aryl, Ure and Glu on day 15, and on the activity of Aryl and Ure on day 30. The application of 100 mg of BPF kg^−1^ d.m. of soil significantly disrupted its homeostasis on day 15 of the experiment. Of the enzymes under analysis, Ure and Glu proved to be the most sensitive to the presence of BPF in soil, which is indicated by the 29% and 18% inhibition of these enzymes activity caused by 100 mg of BPF kg^−1^ d.m. of soil. Similarly, urease proved to be the most sensitive to BPF exposure. This bisphenol also proved to have a beneficial effect on Deh and Pal, whose activity increased 3- and nearly 2-fold compared to the control samples. This relationship also corresponded to the parallel objects on day 15 of the experiment. BPS proved to be the most controversial substitute of BPA. The study results revealed that it inhibited the activity of Pac, Aryl, Glu and Ure while at the same time stimulating the activity of Deh both on day 15 and day 30 of the soil incubation, and Pal on day 30.

**Figure 11:**
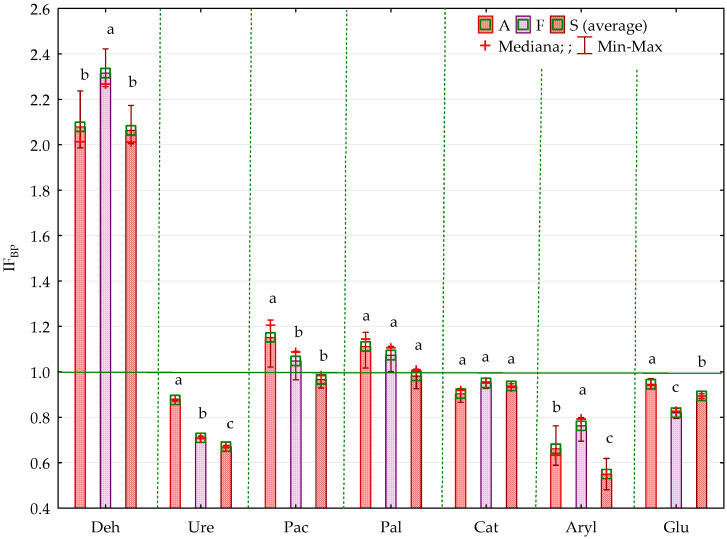
Coefficient of impact (IF_BP_) of bisphenols for enzymes activity in soil contaminated with BPA, BPF and BPS in 15th day of research, A—soil contamination with BPA; F—soil contamination with BPF; S—soil contamination with BPS; Deh—dehydrogenases; Ure—urease, Pal—alkaline phosphatase, Pac—acid phosphatase, Aryl—arylsulphatase, Glu—*β*-glucosidase. Homogeneous groups denoted with letters (a–c) were calculated separately for each enzyme.

**Figure 12:**
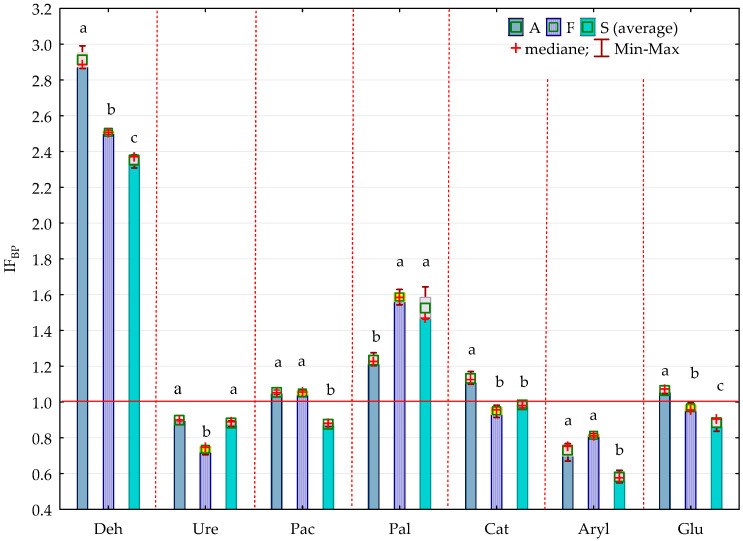
Coefficient of impact (IF_BP_) of bisphenols for enzymes activity in soil contaminated with BPA, BPF and BPS in 30th day of research, Homogeneous groups denoted with letters (a–c) were calculated separately for each enzyme, (for abbreviations see Figure 11).

It is notable that the strength of BPS inhibition was much higher than that of BPA and BPF, which is shown by the homogeneous groups identified in the analysis. The impact factors for each bisphenol (IF_BP_) confirmed the sensitivity of individual enzymes to the applied phenolic compounds (Figure 11, Figure 12). Ure, Aryl and Glu proved to be the most sensitive to pressure from BPA, and Deh the least so. This index emphasised the differences in bisphenol toxicity. Considering the strength of their negative impact on the soil biochemical activity, the phenolic compounds used in this study can be arranged as follows: BPS > BPF > BPA.

## 3. Discussion

### 3.1. Counts and Diversity of Bacteria

The response of microorganisms to the pressure of the phenolic compounds is associated with the activation of a wide range of mechanisms induced by soil microbiome. In the research, only BPA did not stimulate the multiplication of *Pseudomonas* sp. on the 15th day of the study. Consequently, on the 30th day the number of *Pseudomonas* sp. was more than twice as high. BPF and PBS enhanced the multiplication of *Pseudomonas* sp. both on the 15th day respectively by: 71.08% and 24.46% and the 30^th^ day of analysis respectively by: more than twice as high and 86.58%. A similarly large number of mechanisms and, most of all, an impressive pool of enzymes which catalyse bisphenol degradation, were observed in fungi, which is a probable cause of the positive response of these microorganisms to the application of BPA, BPF and BPS to the soil. The count of fungi was particularly stimulating due to BPS. After the application of bisphenol, the number of this group of microorganisms increased as much as twice on the 15th day and three times on the 30th day of analyses. One could expect the *Pseudomonas* sp. count to be stimulated by all the bisphenols. According to the findings of Chuanchuen et al. [78], *Pseudomonas* sp. respond to the substance pressure in soil by activating Resistance-Nodulation-Division (RND) efflux pumps which remove phenols from the cell. They also have a gene (HHDH) encoding halohydrin dehalogenase, which opens the epoxide ring [79]. Moreover, a number of metabolic pathways were identified, which are induced by *Pseudomonas* sp. and which effectively degrade phenolic compounds, including bisphenols [53,54].

Fungi are a reservoir of triphenylmethane reductase, which breaks down phenolic compounds [80], lignin peroxidase, which catalyses their single-electron oxidation [81], polyketide synthase (PKS) and cytochrome P 450, which catalyses monomeric dimerization of phenolic compounds [82]. Reduction of the *Azotobacter* sp. count in soil contaminated with BPA and BPF was not surprising in this study. This is because *Azotobacter* sp. is regarded as a reliable and sensitive indicator of soil contamination with xenobiotics [83,84], although G-negative bacteria, including *Azotobacter* sp., *Arthrobacter* sp. and *Pseudomonas* sp. of phylum *Proteobacteria,* are less susceptible to phenolic compound pressure [85]. These tendencies may be a consequence of a higher isoelectric point (pH = 4–5) for Gram-negative bacteria [86]. More controversy was raised by inhibition of cellulolytic bacteria replication by all the bisphenols under study: BPA, BPF and BPS. However, it turns out that 40% of the sequenced bacteria genomes encode one gene of cellulase, but only 4% are described as proper cellulolytic bacteria [87]. One of the reasons for the negative response of cellulolytic bacteria can be a disruption of the newly discovered strategy of the functional dependence of beneficiaries on helpers, which generates commensalistic and even mutualistic interactions, that provide an opportunity for tangible activity in this group of microorganisms [88]. Also noteworthy are the results of studies in which a beneficial effect of 100 mg BPA on the biomass of microorganisms in wheat-seeded soil was obtained [89]. Nevertheless, the plant is a deposit of microorganisms with phenol degrading genes located in plasmids. In turn, the horizontal transfer of these genetic conditions associated with the plant is favourable in relation to the degradation of volatile contaminants [90].

The CD values for *Actinobacteria* are nearly twice lower than for organotrophic bacteria and the high values of the EP index, ranging from 0.0584 to 0.799 show that although *Actinobacteria* replicate more slowly than organotrophic bacteria [91], they pretend being regarded as microorganisms which effectively biodegrade organic contaminations [92]. This is associated with a large pool of enzymes: proteases, cellulases, amylase, lectinase, catalase, chitinase and urease, which catalyse the process of degradation of complex polymers [93], and with the participation of *Actinobacteria*, in solubilisation of phosphates, siderophores production and nitrogen fixation [92]. The observed succession of the microorganism groups under analysis corresponds to the reports of Sarathchandra et al. [94], according to which fast replicating microorganisms generate higher values of CD regardless of the ecosystem stability and they are called r-strategists, as opposed to slowly replicating or dormant microorganisms, k-strategists. The effect of bisphenols on CD for organotrophic bacteria may be explained by the fact that such microorganisms, such as *Bacillus*, are capable of breaking down BPA. However, bisphenol significantly changes the membrane permeability, sporulation, amino acid and protein expression and metabolism of carbon, purines, pyrimidines and fatty acids (PLFA) [95]. Bisphenol toxicity is associated with indefinite toxicity related to the hydrophobicity of a single compound and the formation of free radicals. Phenols damage the endoplasmic reticulum and mitochondrion. Exposure of microorganisms to bisphenols leads to changes in the properties of the cell membrane, which results in inhibition of respiration and growth of microorganisms and cell lysis [96,97]

Determination of microorganism biodiversity, which is the main factor affecting the soil function has attracted much interest among researchers, which is proven by over 900 papers on the soil metagenome being published before 2017 [98]. The best predictors of soil phylotype abundance include soil pH, climatic factors (temperature, precipitation seasonality) and plant productivity. The most commonly occurring microorganisms include *Alphaproteobacteria, Betaproteobacteria, Actinobacteria, Acidobacteria* and *Planctomycestes* [57]. This study showed that the dominant microorganisms in uncontaminated soil include the phyla: *Actinobacteria, Proteobacteria, Acidobacteria, Chloroflexi, Firmicutes, Planctomyces*. According to Bakker et al. [99], these are the most common phyla in arable lands along with *Bacteroidetes.* Salam and Varma [100] determined the effect of e-waste as a source of phenolic compounds and observed a change in soil bacterial composition towards phylum *Actinobacteria* which overtook *Proteobacteria* and *Firmicutes*. The application to the soil of BPA, BPF and BPS induced corresponding changes in the soil microbiome, significantly enriched by *TM7* in objects with BPF. This was also correlated with the findings described by Siczek et al. [101]. In objects contaminated with a phenolic compound, the authors observed a high abundance of *Actinobacteria* and *Proteobacteria*, with reduced phylum *Chloroflexi*. Similarly, Hassen et al. [102] revealed the presence of α, β and ɣ *Proteobacteria* and *Firmicutes* in soil contaminated with phenolic compounds. Four bacteria types were identified in the author’s research in soil exposed to all the bisphenols: *Nocardioides, Agromyces, Spinghomonas* and *Devosia.* These findings are consistent with those of other researchers. Siczek et al. [101] identified all of these types in soil contaminated with a phenolic compound, except *Spinghomonas.* Tian et al. [103] proposed two phenol degrading strains: *Sphingomonas* sp. PH20 and *Sphingomonas* sp. 31853. It is notable that *Sphingomonas* sp. MV1 was one of the first discovered strains using BPA as a source of carbon and energy, which it obtained in the process of hydroxylation of the phenolic ring and meta-cleavage [104]. Its capability for biodegradation has been confirmed in scientific research many times [101,105]. Hassen et al. [102] and Siczek et al. [101] found bacteria of the genus *Bacillus*, which were also identified in soil contaminated with bisphenols in this study, to be active in decomposing phenolic compounds. According to Li et al. [94], *Bacillus* bacteria are capable of degrading up to 85% of BPA within 24 h. Similarly, Li et al. [106] found the strain *Bacillus* sp. GZB to decompose BPA to p-hydroquinone (HQ) and 4-(2-propanol)-phenol.

### 3.2. Soil Enzyme

Soil enzymes are natural factors which accelerate several soil processes, which gave them the status of indicators of early changes of soil degradation and intensity of biological processes closely connected with the physicochemical properties of the soil environment [46,47]. The high activity of dehydrogenases was expected in line with scientific reports, regardless of the type of bisphenol applied to soil. These expectations stemmed from the participation of dehydrogenases in the conversion of ethylbenzene to 1-phenylethanol and dehydrogenation of phenolic compounds in the presence of dehydrogenases as biocatalysts [107,108]. Bilal et al. [109] report that oxidoreductases, effective in bisphenol biodegradation, are active in a wide range of pH and temperature values. They found a three times higher Deh activity in soil contaminated with BPA and BPF compared to the control sites, which does not correspond to the findings of studies by Zaborowska et al. [110] and Zaborowska et al. [111], in which the correlations were different. Deh proved to be particularly sensitive to BPF, after the application of as little as 5 mg of bisphenol kg^−1^ d.m. of soil. BPS had a similar toxic impact on them. This may be attributed to the fact that stimulation of dehydrogenases activity may also result from the accumulation by microorganisms of lactate dehydrogenase dependent on NAD, which is induced by the demand for a higher energy level following the oxidative stress caused by the pressure of phenolic compounds [112].

A controversy is also raised by a response of acid phosphatase to soil contamination with bisphenols, mainly inhibition of its activity by BPS, whereas the presence of hydroxyl groups in the phenolic ring implies phosphatase adsorption on soil colloids and activates these enzymes. However, given the fact that the process of adsorption may have changed the structure of the function groups, resulting in inhibiting the Pac activity as a consequence of collocation between the enzyme and the substrate, this relationship could be expected [113]. Such concerns are also dissipated by the findings described by Tang et al. [114] who suggest that quinone in soil is formed by phosphorylation of disodium phosphate and the formation of phenol, catalysed by phosphatases. Quinones, in turn, are regarded as important inhibitors of enzymatic activity. Together with the SH group in cysteinyl, transformed to SS bonds, they reduce the urease activity [115]. The toxicity of quinones is associated with arylation of the thiol group in Cys. 1,4 and covalent modification. Currently, the group of urease inhibitors has been expanded to include hydroquinone, derivatives of coumarin, phenolic aldehydes and catechol [116]. Catechol inactivates the urease metallocentre by the formation of catechol-metal complexes. It is also oxidised to form orto-benzoquinone, which is a strong urease inhibitor as a result of the modification of the functional groups in the enzyme protein [117,118]. The effectiveness of urease activity inhibition is also correlated with the presence and position of substituents in phenolic compounds [119]. They are probably caused more by methoxyl and hydroxyl groups in phenolic organic compounds, as opposed to nitro groups which exhibit low-protein inhibition of urease activity [120,121]. Due to their structure with two ortho hydroxyl groups, isoflavone polyphenols become less toxic to urease only after the c-isoflavone ring is broken [122]. It should be emphasised that binding substituents with single pairs of electrons to the phenyl ring around the thiourea core and the presence of fluorine atom in phenyl groups, regardless of its position, also results in strong urease inhibition [123,124]. The findings of Zaborowska et al. [110,111] and Siczek et al. [101] corroborate the experiment results. Both BPS and BPF proved to be significant inhibitors of both Pac and Ure. The findings related to Glu activity were more debatable. BPF and BPS had a negative effect on the Glu activity on day 30 of the study. This is probably a consequence of low kinetic parameters, such as maximum reaction rate (V_max_) and Michaelis–Menten constant (K_m_), which generate Glu adsorption at the site of binding with hydroxyl phenol [112]. This thesis can be partly corroborated by the findings of Zaborowska et al. [111], who found the response of Glu to BPF was similar, while the application of BPS did not generate such low enzyme sensitivity to the phenolic compound. A phenolic compound was also a significant Glu inhibitor according to Siczek et al. [101].

The most important factors shaping the microbiological response to bisphenols are: genes encoding enzymes responsible for catabolism of bisphenols located in bacterial chromosomes or degrading plasmids, functional viability of enzymes different in organic and mineral soils, desorption and adsorption of phenols in soil affecting their mobility and availability as well as organic matter and pH. In addition, the intensity of phenolic compound inhibition is significantly affected by the presence and position of selected substituents It is also important to be aware of the diverse degradative activity of bacteria, and in particular the activation of various metabolic pathways under both aerobic and anaerobic conditions.

The changes in the reaction of microorganisms and the activity of individual soil enzymes, that were observed in this study, were a response to a biotic stress caused by soil contamination with bisphenols. The diverse reaction of microorganisms and enzymes to BPA, BPF and BPS that was observed in this study is likely due to the fact that enzymes can be both extracellular and intracellularly metabolized by microbes. In the soil environment the enzymes react with minerals, are broken down by proteolytic enzymes or undergo thermal denaturation [125]. It should be emphasized that phenolic compounds are a growth medium for microorganisms, a source of carbon and energy. However, the condition for using these substrates is the presence of hydroxyl groups equivalent to the presence of molecular oxygen as a co-substrate [126].

## 4. Materials and Methods

### 4.1. Soil and Facilities

The experiment was conducted with proper brown soil classified as loamy sand, which had a granulometric composition that was determined following the international soil classification for soil nomenclature and creating legends for soil maps [125,127]. The soil was collected from the genetic horizon Ap, at the Teaching and Experiment Centre in Tomaszkowo, developed agriculturally for cereal cultivation, situated in the Olsztyn Lakeland. This area is dominated by Eutric Cambisol soils formed on sand and loam. The Olsztyn Lakeland (Pojezierze Olsztyńskie) is the largest mesoregion in the Mazurian Lake District in the East European Lowland in the temperate warm transitional climate zone with an average annual temperature of 7.2 °C. A full characterisation of the soil material, with its granulometric composition and selected physicochemical, biochemical and microbiological properties is provided in Table 1, which was determined using the methodology described by Borowik et al. [126,128].

### 4.2. Experiment Setup

The choice of the key experiment stages was based on the fact that there is scarce data on the effect of BPA, BPF and BPS, on both the biochemical and microbiological activity of soil. Therefore, variable factors which could potentially modify the expected analysis results were eliminated and the experiment was conducted in a laboratory, under strictly controlled ex situ conditions. To determine ultimately which of the bisphenols disrupts the soil homeostasis to the greatest extent and mainly to emphasise the difference in toxicity of the proposed phenolic compounds, one level of soil contamination with bisphenols was applied—100 mg BP kg^−1^ d.m. of soil. The assays were performed against control objects, uncontaminated with bisphenols. Since bisphenols are poorly soluble in water, adding them to air-dry soil was preceded by dissolving each bisphenol in ethanol at the ratio of 3:1 (ethanol:bisphenol). The experiment was conducted in 150 cm^3^ glass beakers in three replicates. The soil samples (100 g d.m.) were prepared separately for the two experiment runs. After being thoroughly homogenised and their moisture content stabilised at 50% of the capillary water capacity, the soil samples were incubated at the constant temperature of 25 °C for 15 and 30 days. The soil moisture content was monitored throughout the experiment.

### 4.3. Characteristics of Bisphenols

**Figure 13:**
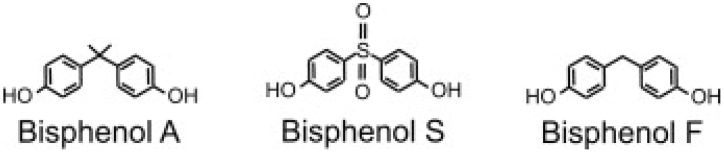
Chemical structure of bisphenols A, S and F.

The research dealt with three bisphenols (Figure 13). Referring to the safety data sheet issued by Sigma–Aldrich, (Poznań, Poland) bisphenol A (BPA) (synonyms: 4,4′-isopropylidenediphenol; 2,2-bis(4-hydroxyphenyl)-propane), bisphenol F (BPF) (synonyms: 4,4′-methylenediphenol, bis(4-hydroxyphenyl) methane and bisphenol S (BPS) (synonyms: 4,4′-sulfonyldiphenol, 4-hydroxyphenyl sulfone, bis(4-hydroxyphenyl) sulfone) were used.

All the bisphenols were white crystalline substances with the purity of ≥98.0% (HPLC - high-performance liquid chromatography). Selected physical and chemical properties of the bisphenols are shown in Table 4. The level of soil contamination with BPA, BPF and BPS was determined based on two guidelines. First, legal guidelines, according to which the permissible bisphenol concentration at the depth of 0–0.3 m was 0.1 mg kg^−1^ in soil which is classified as agricultural land, excluding built up and urban areas [129]. The second guideline was established based on the need to determine the level of potentially negative bisphenol impact (lower than that analysed in the experiments conducted so far) common to the selected phenolic compounds [110,111].

### 4.4. Sample Analysis

On days 15 and 30 of the experiment, the organotrophic bacteria (Org), Actinobacteria (Act) and fungi (F) count was determined with the serial dilution method, in three replications. The composition of the microbiological media was as follows: organotrophic bacteria (Bunt and Rovira medium), Actinomycetes (Parkinson medium) and fungi (Martin medium). Microbial counts were performed according the media and procedure described by Borowik et al. [128]. Colony-forming units (cfu) were counted on 10 consecutive days with a colony counter. The results provided grounds for determining the colony development index (CD) [94] and the ecophysiological diversity index (EP) [131] from the following formulas:

(1) $$CD=[(\frac{N_{1}}{1}+\frac{N_{2}}{2}+\frac{N_{3}}{3}\cdots \cdots \frac{N10}{10})\;\cdot \;100]$$

where N_1_, N_2_, N_3_,...N_10_—sum of ratios of the colony numbers identified on each day (1, 2, 3,...10) and the sum of all the colonies identified during the entire experiment and:

EP = −Σ(pi × log pi)
(2)

where pi denotes the number of microbe colonies replicated on a specific day, divided by the number of all the colonies.

To consider the bisphenol effect on the soil microbiological activity in a broader perspective, the count of eight microorganism groups was also determined. The composition of the microbiological media was as follows: oligotrophic bacteria (Olig) and copiotrophic bacteria (Cop) (Ohta and Hattori medium), cellulolytic bacteria (Cel), nitrogen immobilising bacteria (Im) and ammonification bacteria (Am) (Winogradski medium), *Pseudomonas* sp. (Ps) and *Arthrobacter* sp. (Art) (Mulder and Antheumisse medium), *Azotobacter* sp. (Az) (Fenglerowa medium). The determination was performed by serial dilutions, in three replicates. The microorganisms were cultured on Petri dishes on microbiological medium whose composition and preparation procedure were described by Borowik et al. [128]. All the microorganism groups were incubated at a constant temperature of 28 °C.

### 4.5. DNA Extraction and Bioinformatic Analysis of Bacteria Taxons

Isolation of genomic DNA from a soil sample was based on a modified method utilising the “Genomic Mini AX Bacteria +” kit (A&A Biotechnology). Determination of bacterial DNA was preceded by mechanical lysis of the samples with zirconium balls in a FastPrep-24 device and additional purification in an Anti-Inhibitor Kit. The presence of bacterial DNA in the soil samples was determined by the. fluorometric method on a Qubit 4 Fluorometer server. Real-time PCR was performed with SYBR Green dye. Preliminary denaturation was carried out for 3 min at 95 °C and denaturation was carried out for 15 s at 95 °C. Universal starters: 1055F (5′-ATGGCTGTCGTCAGCT-3′) and 1392R (5′-ACGGGCGGTGTGTAC-3′) which amplify a fragment of the bacterial gene 16S RNA were bound during 30 s at 58 °C. The PCR product was extended for 30 s at 72 °C. The melting curve of the PCR product was determined by measurement fluorescence in the temperature range 65 °C–>95 °C. Sequencing of the gene encoding amplicon 16S sequences was performed based on the complementarity determining region V3-V4. The bioinformatic analysis was performed on the MiSeq sequencer of the QIIME software package based on the GreenGenes v13_8 reference sequence database. Sequencing performed by Genomed SA (Warsaw, Poland) enabled the reading classification down to the genus level. The sequencing readings on the amplicon of gene 16S RNA were assigned with OTU in accordance with the taxon in the manner described by Ravel et al. [132].

### 4.6. Determination of Soil Enzyme Activity

The biochemical analyses were performed in three replicates, simultaneously with the microbiological analyses. The activity of the following enzymes was determined in 1 kg of soil: dehydrogenases (Deh) (EC 1.1), urease (Ure) (EC 3.5.1.5), acid phosphatase (Pac) (EC 3.1.3.2), alkaline phosphatase (Pal) (EC 3.1.3.1), arylsulphatase (Aryl) (EC 3.1.6.1), *β*-glucosidase (Glu) (EC 3.2.1.21) and catalase (Cat) (EC 1.11.1.6). The activity of the six enzymes was determined by measuring the extinction of the obtained reaction product with a Perkin-Elmer Lambda 25 spectrophotometer (Massachusetts, USA). Catalase activity was determined by titration. The calculated biochemical indices were expressed in the following units: dehydrogenases—μmol TFF (triphenyl formazan) kg^−1^ d.m. of soil h^−1^, urease—mmol N-NH _4_ kg^−1^ d.m. of soil h^−1^, acid phosphatase, alkaline phosphatase, arylsulphatase and *β*-glucosidase - mmol PN (p-nitrophenol) kg^−1^ d.m. of soil h^−1^, catalase—mol O_2_ kg^−1^ d.m. of soil h^−1^. The substrates were characterised and detailed procedures of the biochemical analyses were described by Borowik et al. [128].

### 4.7. Statistical Data Analysis and Methodology of Calculations

The experiment results were configured based on the statistical analyses performed in the Statistica 13.1 package [133]. The η^2^ coefficient of percentile variability of the variable under study was determined by an ANOVA analysis of variance. A multidimensional analysis, PCA, was applied to determine the impact of BPA, BPF and BPS on the colony development index (CD) and the ecophysiological diversity index (EP). Tukey’s test at *p* = 0.01 was used to determine homogeneous variances between soil enzymes and microorganism groups. The differences in their responses to soil contamination with BPA, BPF and BPS were emphasised by showing the fluctuating tendencies with the bisphenol impact factor (IF_BP_), calculated from the formula:

(3) $${\mathrm{IF}}_{\mathrm{BP}}=\frac{A_{\mathrm{BP}}}{A_{C}}$$

Where IF_BP_—the factor of the impact of increasing bisphenol (BP) soil contamination levels, (IF_BP_ < 1—inhibition of the enzyme activity and groups of microorganisms by BPA, BPF and BPS, IF_BP_ > 1—stimulation of the soil enzyme activity and groups of microorganisms by BPA, BPF and BPS; A_BP_—enzyme activity and groups of microorganisms in the soil subjected to the increasing BPA, BPF and BPS contamination pressure; A_C_—activity of the enzyme and groups of microorganisms in the control soil non-contaminated with BPA, BPF and BPS.

Visualisation of the genome data only with the sequences exceeding 1% was performed with the following statistical analyses: gplots library [134], a bilateral test of statistical hypotheses—G-test (w/Yates’) + Fisher’s, with the interval confidence method Asymptotic with CC [135] configured in the STAMP 2.1.3. software, the thermal map based on the RStudio v1.2.5033 software [136], system R v3.6.2 [137]. The data were presented in a circular arrangement with Circos 0.68 software (Canada’s Michael Smith Genome Sciences Center, Vancouver, British Columbia V5Z 4S6, Canada).

## 5. Conclusions

Bisphenols A, F and S interfered significantly in the soil microbiome and disrupt its homeostasis. Bisphenol F (BPF) was regarded as the most toxic, followed by BPS and BPA. Cellulolytic bacteria proved to be the most sensitive to soil contamination with bisphenols. All of the phenolic compounds stimulated the count of *Pseudomonas* bacteria and the fungi colony growth rate and count. The bisphenols applied to soil played an equally important role in moulding its genetic diversity. BPA had the least significant effect on this parameter. *Actinobacteria* and *Proteobacteria* species were found to dominate in uncontaminated soil and in that exposed to BPA. BPF and BPS reduced the abundance of *Proteobacteria* and *Acidobacteria* and increased that of *Actinobacteria*. Contamination of soil with BPA, BPF and BPS significantly modified its enzymatic activity. It was inhibited the most strongly by BPS, and the least strongly by BPA. Considering the enzyme sensitivity to each bisphenol, the following sequences can be proposed: on day 15: BPA: Aryl > Ure > Cat > Glu > Pal > Pac > Deh; BPF: Ure > Aryl > Glu > Cat > Pac > Pal > Deh, BPS: Aryl > Ure > Glu > Cat > Pac > Pal > Deh; on day 30: BPA: Aryl > Ure > Glu > Cat > Pac > Pal > Deh; BPF: Ure > Aryl > Glu = Cat > Pac > Pal > Deh, BPS: Aryl > Pac = Ure = Glu > Cat > Pal > Deh.

## Figures and Tables

**Table 1:** Soil characteristic (physicochemical, chemical, biochemical and microbiological properties).

Parameters	Unit	Value	References
Granulometric Composition of fractions (%) (*d*,mm)	sand 2.00 ≥ *d* > 0.05	74.93	[62]
	silt 0.05 ≥ *d* > 0.002	22.85	
	clay *d* ≤ 0.002	2.22	
pH_KCl_	(mol KCl dm^−3^)	6.70	[63]
HAC	(mM (+) kg^−1^ dry matter of soil)	11.40	
EBC		49.00	[64]
CEC		0.40	
BS	(%)	81.10	[65]
C_org_	(g kg^−1^ dry matter of soil)	9.30	
N_tot_		0.62	[66]
P_aveilabe_	(mg kg^−1^ dry matter of soil)	93.68	[67]
K_aveilabe_		141.10	
Mg_aveilabe_		42.00	[68]
	Enzymes activity		
Deh	(μmol triphenyl formazan (TFF) kg^−1^ dry matter of soil h^−1^)	0.546	[69]
Ure	(mmol N-NH_4_ kg^−1^ dry matter of soil h^−1^)	0.632	[70]
Cat	(mol O_2_ kg^−1^ dry matter of soil h^−1^)	0.086	
Pal	(mmol 4-nitrofenol (PN) kg^−1^ dry matter of soil h^−1^)	2.309	
Pac		0.510	
Aryl		0.013	
Glu		0.422	
	Number of microorganisms		
Org	(cfu 10^*n*^ kg^−1^ dry matter of soil)	27.971	[71]
Act		116.194	[72]
F		802.414	[73]
Ps		33.473	[74]
Art		37.657	
Cel		27.197	[75]
Olig		191.771	
Cop		166.667	
Im		103.208	[76]
Am		341.702	
Az		20.921	[77]

HAC—hydrolytic activity, EBS—sum of its total exchangeable base cation, CEC—exchangeable capacity of the sorption complex, BS—soil saturation with cations; Deh—dehydrogenases; Ure—urease, Pal—alkaline phosphatase, Pac—acid phosphatase, Aryl—arylsulphatase, Glu—*β*-glucosidase; Org—organotrophic bacteria, Act—Actinomycetes, F—mold fungi, Ps—*Pseudomonas* sp., Art.—*Arthrobacter* sp., Cel—cellulolytic bacteria, Olig—oligotrophic bacteria, Cop—copiotrophic bacteria, Im—nitrogen immobilizing bacteria, Am—ammonification bacteria, Az—*Azotobacter* sp., *n*-exponent: 7 for Org, Act, Ps, Art., Cel, Olig, Cop, Im, Am; 5 for F; 3 for Az.

**Table 2:** The number of microorganisms in soil contaminated with BPA, BPF and BPS on the 15th and 30th day of the research, (cfu 10*^n^*kg^−1^ d.m. of soil).

Kind of BP	Org	Act	F	Ps	Art	Cel	Olig	Cop	Im	Am	Az
15th day											
C	470.874 ^a^	482.581 ^b^	87.664 ^c^	109.973 ^c^	145.620 ^a,b^	169.132 ^a^	910.884 ^b^	242.700 ^b^	1305.271 ^a^	847.175 ^a^	139.553 ^a^
A	330.097 ^b^	464.306 ^b^	147.630 ^a^	81.356 ^d^	85.122 ^c^	165.725 ^a^	767.608 ^d^	223.729 ^b^	381.168 ^d^	566.478 ^b^	164.218 ^a^
F	348.372 ^b^	441.462 ^b^	115.934 ^b,c^	187.547 ^a^	188.307 ^a^	67.578 ^b^	991.648 ^a^	302.961 ^a^	647.684 ^b^	934.700 ^a^	29.613 ^b^
S	245.288 ^c^	539.406 ^a^	147.915 ^a^	136.434 ^a,b^	136.434 ^b^	59.690^c^	834.884 ^c^	333.333 ^a^	491.473 ^c^	865.116 ^a^	31.008 ^b^
30th day											
C	297.783 ^b^	254.561 ^d^	103.564 ^d^	68.057 ^d^	133.919 ^a^	164.654 ^a^	488.108 ^c^	283.937 ^b^	381.998 ^c^	607.391 ^b^	150.018 ^b,c^
A	318.271 ^b^	581.813 ^a^	216.952 ^c^	176.448 ^a^	169.544 ^a^	104.334 ^b^	637.514 ^b^	333.717 ^a^	601.458 ^a^	817.031 ^a^	119.678 ^c^
F	252.315 ^b^	435.307 ^c^	271.401 ^b^	169.197 ^b^	42.661 ^c^	97.614 ^b^	624.729 ^b^	185.105 ^d^	436.732 ^b^	399.855 ^c^	245.842 ^a^
S	382.543 ^a^	457.480 ^b^	305.641 ^a^	126.984 ^c^	80.527 ^b^	80.527 ^c^	744.870 ^a^	239.257 ^c^	552.846 ^a^	651.955 ^b^	160.279 ^b^

n—exponent: 7 for Org, Act, Ps, Art., Cel, Olig, Cop, Im, Am; 5 for F; 3 for Az; Homogeneous groups denoted with letters (a–d) were calculated separately for each group of microorganisms, C—control, A—soil contaminated with bisphenol A, F—soil contaminated with bisphenol F, S—soil contaminated with BPS, (for abbreviations see Figure 1).

**Table 3:** Enzymatic activity in soil contaminated with BPA, BPF, BPS in 15th and 30th day of research, kg^−1^ d.m. of soil h^−1.^

Kind of BP	Deh	Cat	Pac	Pal	Aryl	Glu	Ure
	(μMol TFF)	(mol O_2_)	(mmol 4-Nitrophenol PN)				(mmol N-NH_4_)
15th day							
C	6.625 ^c^	0.422 ^a^	1.606 ^b^	2.159 ^b,c^	0.060 ^a^	1.115 ^a^	2.435 ^a^
A	13.743 ^b^	0.381 ^c^	1.845 ^a^	2.392 ^a^	0.040 ^b,c^	1.051 ^b^	2.142 ^b^
F	15.320 ^a^	0.401 ^b^	1.679 ^a,b^	2.312 ^a,b^	0.046 ^b^	0.914 ^d^	1.731 ^c^
S	13.648 ^b^	0.396 ^b,c^	1.560 ^b^	2.115 ^c^	0.033 ^c^	0.999 ^c^	1.634 ^d^
30th day							
C	5.233 ^d^	0.379 ^b^	1.683 ^b^	1.892 ^c^	0.049 ^a^	0.994 ^a,b^	2.224 ^a^
A	15.241 ^a^	0.418 ^a^	1.774 ^a^	2.336 ^b^	0.036 ^c^	1.050 ^a^	1.933 ^b^
F	13.121 ^b^	0.360 ^b^	1.768 ^a^	3.000 ^a^	0.040 ^b^	0.951 ^b^	1.630 ^c^
S	12.311 ^c^	0.373 ^b^	1.473 ^c^	2.884 ^a^	0.028 ^d^	0.873 ^c^	1.852 ^b^

Homogeneous groups specified in columns, for each enzyme, depending on the increasing doses of BPA, BPF and BPS during the 15 and 30 days of experiment. Deh—dehydrogenases; Ure—urease, Pal—alkaline phosphatase, Pac—acid phosphatase, Aryl—arylsulphatase, Glu—*β*-glucosidase. Homogeneous groups denoted with letters (a–d) were calculated separately for each enzyme, C—control; A—soil contaminated with BPA; F—soil contaminated with BPF; S—soil contaminated with S.

**Table 4:** Selected physicochemical properties of BP.

Compound	Molecular Formula	Molecular Weight	BCF	VP	Log K_ow_	WS	BP (°C)
Bisphenol A	C_15_H_16_O_2_	228.29	71.85	2.27 × 10^−7^	3.64	172.7	363.54
Bisphenol S	C_12_H_10_O_4_S	250.27	36.97	4.72 × 10^−10^	1.65	351.8	422.52
Bipshenol F	C_13_H_12_O_2_	200.23	34.73	3.72 × 10^−7^	3.06	542.8	351.92

BCF—bioconcentration factor, VP—vapor pressure; predicted data (at 25 °C, mm Hg), WS—water solubility; predicted data (at 25 °C, mg dm^−3^), BP—boiling point [130].
